# Case Report: Diagnosis and treatment report and literature review of 2 cases of VHL-deficient renal cell carcinoma

**DOI:** 10.3389/fonc.2026.1645910

**Published:** 2026-07-21

**Authors:** Yanchen Wang, Tongbin Gao, Na Ren, Yuxuan Liu, Qingxuan Li, Xiaoyan Guo

**Affiliations:** 1 Department of Urology, Weifang people’s Hospital, Shandong Second Medical University, Weifang, Shandong, China; 2 School of Nursing, Shandong Second Medical University, Weifang, Shandong, China; 3 School of Clinical Medicine, Shandong Second Medical University, Weifang, Shandong, China; 4 Department of Nuclear Medicine, Weifang people’s Hospital, Shandong Second Medical University, Weifang, Shandong, China

**Keywords:** hypoxia-inducible factor, screening test, targeted immunotherapy drugs, VHL disease, VHL-deficient renal cell carcinoma

## Abstract

Von Hippel-Lindau (VHL) disease is a rare familial autosomal dominant disorder with an incidence rate of approximately 1 in 36,000. It primarily results from mutations or inactivation of the VHL tumor suppressor gene located on chromosome 3p25-p26. The hallmark of this disease is hereditary hemangioblastoma, which can affect multiple organs and systems, including the brain (commonly infratentorial), spinal cord, retina, and internal organs such as the kidneys, adrenal glands, and pancreas. Less commonly, lesions may include papillary cystadenomas and endolymphatic sac tumors (ELST), which can form in the epididymis or broad ligament. The leading causes of death in these patients are hemangioblastomas and renal cell carcinoma of the central nervous system. Due to the multidisciplinary nature of the disease, diagnosis and management require a multidisciplinary team (MDT) consultation and collaboration among various specialties. Clinical diagnosis, genetic implications, and prognosis must be assessed comprehensively, with genetic testing confirming the diagnosis. Cases of VHL are exceptionally rare in clinical practice, and there remains a significant unmet need for effective treatments, particularly in rare tumors. Misdiagnosis and mistreatment are common, and repeated surgical interventions can exacerbate kidney damage. Early and accurate diagnosis, followed by proactive treatment, can significantly improve prognosis. Recently, our department admitted two patients with VHL-deficient renal cell carcinoma. Through surgery, radiofrequency ablation, and subsequent targeted therapy, the therapeutic outcomes were highly favorable. This report introduces the treatment of these two cases and provides a literature review on the current progress in the diagnosis, treatment, and prognosis of VHL-deficient renal cell carcinoma. Additionally, it discusses data on the screening of VHL patients and their close relatives, while emphasizing the optimization of individualized management for renal cell carcinoma to enhance the understanding, diagnosis, and treatment of the disease.

## 1 Introduction

Renal cancer, the third most common malignancy in the urinary system, has a high malignancy potential, It is estimated that in 2022, there were approximately 77,410 new cases of kidney cancer and about 46,345 deaths from the disease in China, The global distribution of kidney cancer is uneven, with the incidence rate in developed countries being significantly higher than that in developing countries. Moreover, the incidence of RCC is higher in men than in women (the ratio is 1.5:1), and the mortality rate among men is also higher than that among women. The cause of renal cell carcinoma (RCC) is still unclear. Smoking and obesity are currently recognized as risk factors for kidney cancer. Other potential risk factors include high blood pressure, antihypertensive drugs, lesion location, and race (1–7).particularly when bilateral renal tumors are present, necessitating consideration of Von Hippel-Lindau (VHL) disease. Research indicates that 70% of patients with VHL disease develop renal cell carcinoma (RCC) (8).VHL syndrome is a rare autosomal dominant genetic tumor disorder. Its incidence rate is 1 in 36,000, and the average age of onset is 26.2 to 30.8 years. The natural lifespan is before the age of 50 (9–11).The VHL gene, a tumor suppressor gene, plays a critical role in kidney development. Mutations (or deletions) of the VHL gene lead to the activation of hypoxia-inducible factors (HIFs), which in turn alter downstream growth factors, promoting tumor growth. VHL-associated lesions can affect multiple organs, with renal cell tumors being the leading cause of mortality. Retrospective studies on familial VHL diseases suggest that the median survival for these patients ranges from 60 to 67 years (12). RCC associated with VHL gene mutations progresses more slowly compared to sporadic RCC, making early diagnosis, prompt surgery, and subsequent targeted therapy essential for a favorable prognosis. Timely comprehensive evaluation and ongoing treatment, including follow-up, are essential to patient management.

## 2 Case report: 1

A 45-year-old female patient was admitted in 2021 after bilateral renal tumors were detected. She presented with no symptoms such as low back pain, hematuria, or systemic signs like loss of appetite, fatigue, or weight loss. Upon admission, contrast-enhanced CT of the lower abdomen and pelvis revealed bilateral renal tumors, suggesting a high likelihood of renal cancer, as well as bilateral renal cysts and pancreatic cystadenoma. Cranial MRI identified an intraspinal space-occupying lesion at the atlas level. A biopsy of the left renal lesion showed abnormal cell nests, some glandular in arrangement, and others with clear cytoplasm, consistent with clear cell renal carcinoma based on immunohistochemistry. Genetic testing identified a VHL mutation (Chr3:10191590; c.585_586delGA), which was inherited.

Genetic testing of the patient’s parents showed no VHL mutations. However, her son tested positive for the same mutation (c.585_586delGA, resulting in a frameshift mutation p.K196Rfs*59), classified as pathogenic according to ACMG guidelines. No VHL mutation was found in the patient’s daughter.

**Figure 1:**
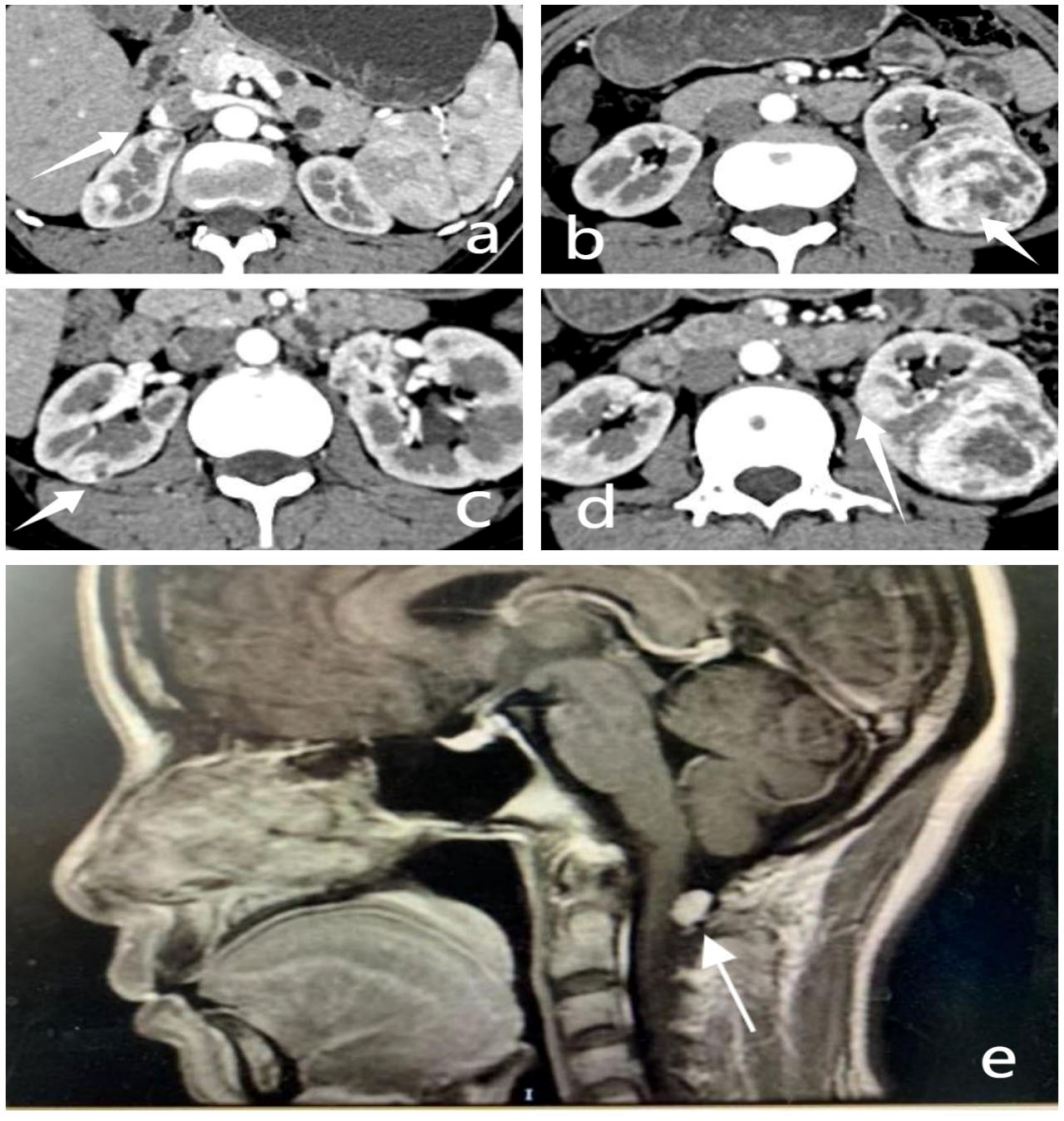
**(A–D)** The white arrows indicate the locations of renal cell carcinomas. Among them, the renal cell carcinoma in the left proximal lumbar region is the largest [shown in **(B)**], with an approximate diameter of 5×6 cm. **(E)** Horizontal segment of the atlas—an intraspinal tumor. Graph timeline **(A–D)**: 2021/8/3.

**Figure 2:**
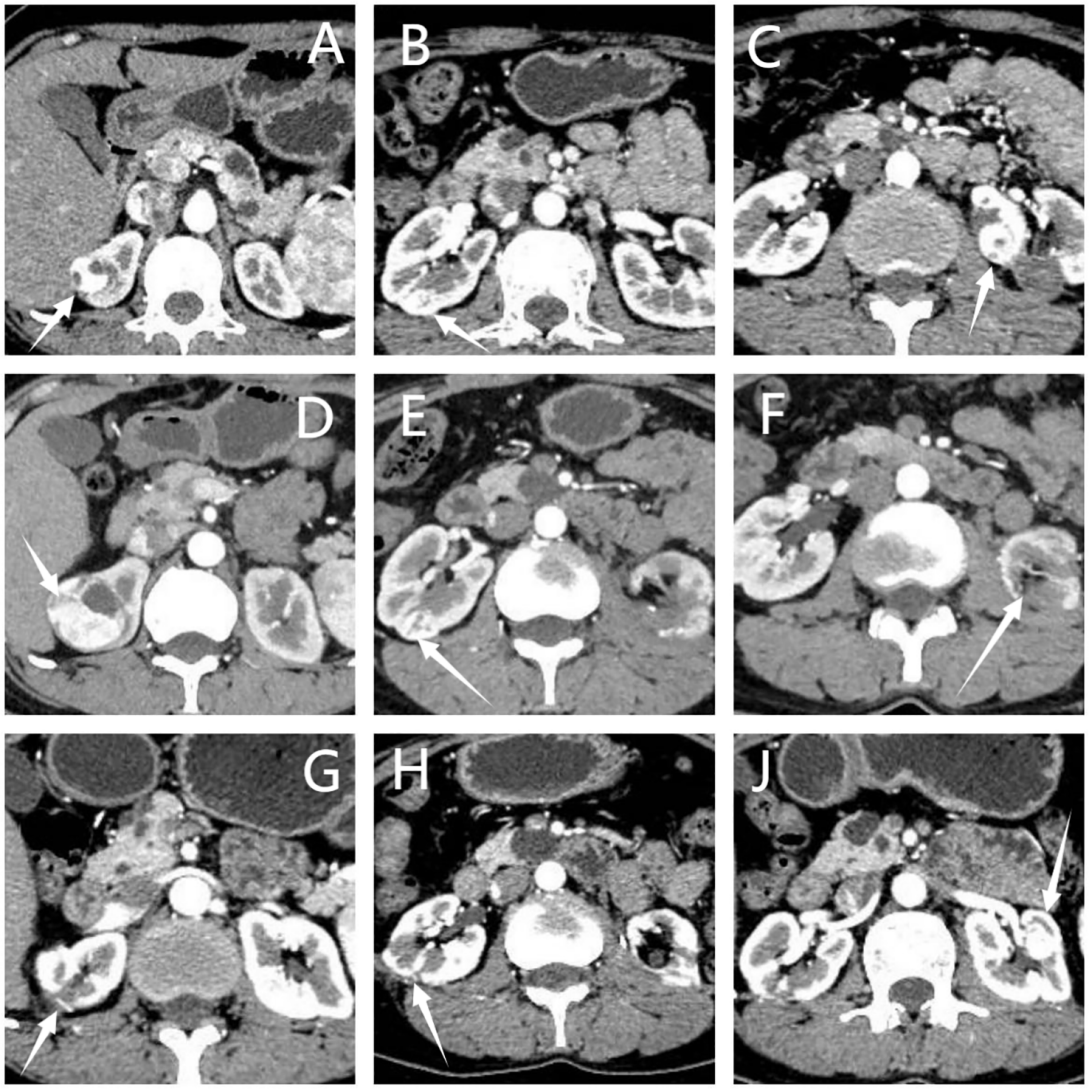
**(A–C)** First follow-up examination one month after radiofrequency ablation (RFA) of the left renal tumor. **(D–F)** Laparoscopic left renal tumor resection performed in the second stage, 6 months after RFA. **(D)** The tumor on the right side is slightly larger than before. At this stage, pazopanib targeted drug therapy was recommended for the patient. Three months after starting medication, laparoscopic resection of the right renal tumor was performed in the first stage. **(G–J)** Re-examination data one year after the right kidney tumor surgery, demonstrating very favorable postoperative results. The patient has been continuing oral pazopanib therapy, and follow-up is ongoing. Graph timeline **(A–C)**: 2021/12/14. Graph timeline **(D–F)**: 2022/06/05. Graph timeline **(G–J)**: 2023/09/18.

Following multidisciplinary team (MDT) consultation, a treatment plan was established: radiofrequency ablation (RFA) was performed for stage I left RCC (larger tumor), while laparoscopic partial nephrectomy (PN) was carried out for stage II left RCC and stage I right RCC. The patient was also treated with pazopanib targeted therapy. After four years of regular follow-up, the patient’s condition has shown significant improvement, with the tumors well controlled and no signs of metastasis, as illustrated in **Figures 1**, **2**.

## 3 Case report: 2

The patient is a 32-year-old male who presented with a complaint of painless gross hematuria lasting for one month, without associated symptoms such as low back pain. Upon admission, an enhanced CT scan of the lower abdomen and pelvic cavity revealed multiple pancreatic cysts, along with enhanced nodules within the pancreas, suggesting the coexistence of pancreatic cysts and tumors. Additionally, both kidneys showed signs of polycystic kidney disease, with enhanced nodules detected in both kidneys, raising suspicion for renal cancer. Given the patient’s medical history and the findings in the pancreas and kidneys, a diagnosis of VHL disease was considered. Post-lumbar spine surgery, the patient developed atrophy in the right iliac muscle, iliopsoas muscle, right hip joint, and muscles surrounding the right thigh.

The patient’s medical history includes hypertension and type 2 diabetes. Surgical history includes cerebellar tumor resection in 2009, followed by adjuvant gamma knife treatment. In 2012, a lumbar tumor resection was performed, and a subsequent cerebellar tumor resection with gamma knife treatment was conducted in 2019.

No significant positive findings were found during the abdominal physical examination.

Preliminary diagnosis: VHL-deficient RCC, polycystic kidney disease, and a history of lumbar and cerebellar tumor surgeries.

**Figure 3:**
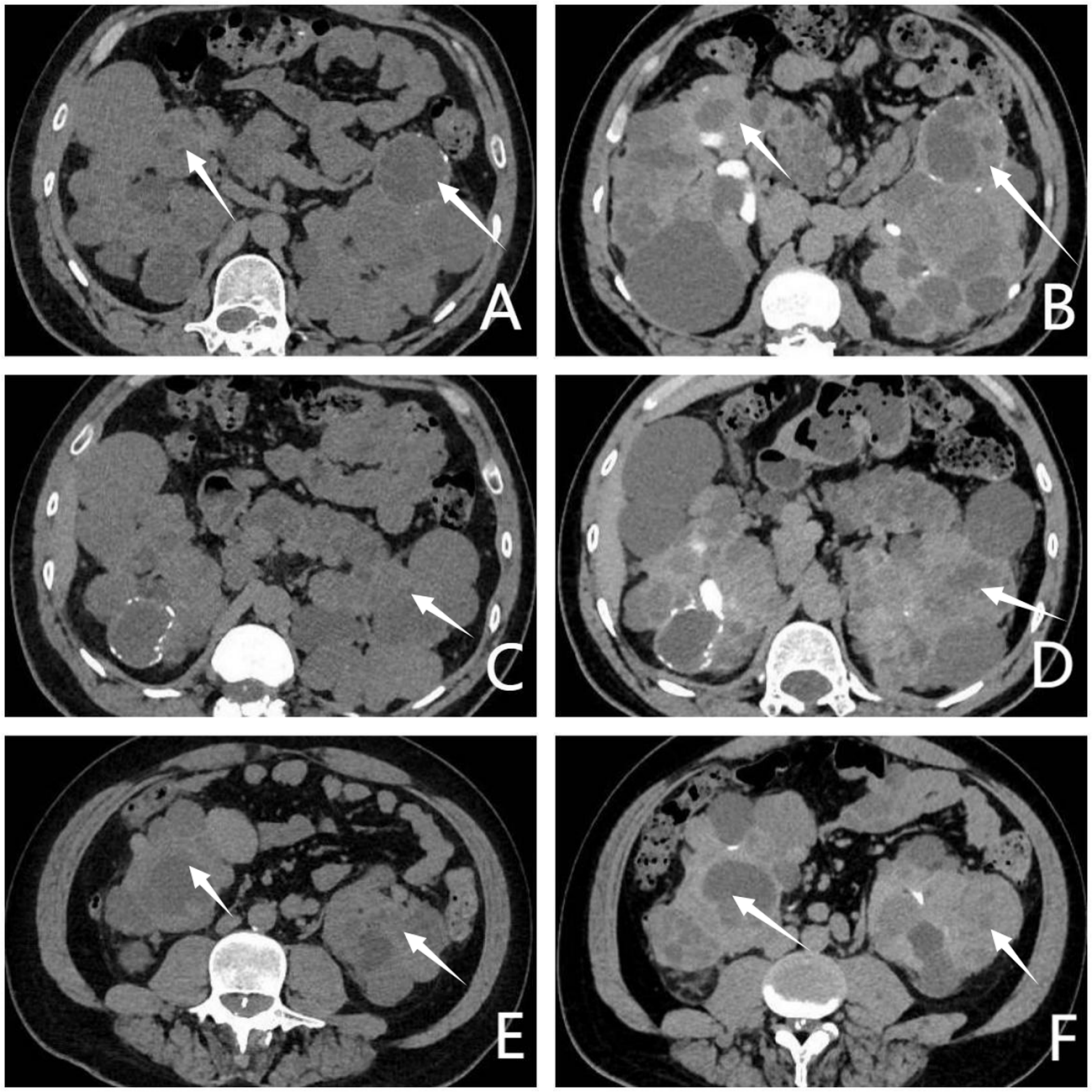
**(B, D, F)** Baseline imaging data before the first use of the targeted drug pazopanib. The patient was diagnosed with polycystic kidney disease combined with bilateral renal cell carcinoma, accompanied by hematuria. After confirming VHL-deficient renal cell carcinoma, surgery was not deemed advisable. Instead, the patient received comprehensive treatment with pazopanib. Three months after starting the medication, the first re-examination imaging **(A, C, E)** demonstrated excellent therapeutic results. The patient continues to take oral pazopanib, with ongoing follow-up. Graph timeline **(B, D, F)**:2024/12/17. Graph timeline **(A, C, E)**:2025/03/24.

The patient was started on oral pazopanib (800 mg) and has been under regular follow-up for 10 months, as shown in **Figure 3**.

Genetic testing revealed a heterozygous mutation in the VHL gene, designated NM_000551.4:c.340 + 2T>A. This mutation occurs at the second base downstream of exon 1 of the VHL gene, disrupting the donor or recipient splicing site and typically leading to the loss of protein function.

## 4 Discussion

The VHL gene is named after VHL disease, a rare autosomal dominant genetic disorder caused by the inactivation of the VHL tumor suppressor gene. The clinical diagnosis of VHL disease is based on several key manifestations: hemangioblastomas (in the central nervous system or retina), RCC, pheochromocytoma, multiple pancreatic cysts or neuroendocrine tumors, and endolymphatic sac tumors. A clinical diagnosis of VHL can be made when the following conditions are met (1): a clear family history, with the presence of one of the above seven tumors; or (2) in the absence of a family history, the presence of at least two hemangioblastomas or one hemangioblastoma plus any one of the above seven tumors (9, 13).

The VHL tumor suppressor gene, located on the short arm of chromosome 3 (3p25-p26), plays a critical role in kidney growth and development. Mutations in this gene lead to the activation of HIF, which cause alterations in downstream growth factors, promoting tumor growth. This mutation is particularly associated with the development of sporadic clear cell RCC (ccRCC). While renal cysts and RCC can occur independently, the combination of renal cysts and RCC is a hallmark of VHL disease and is considered a key diagnostic feature. Renal tumors are the leading cause of mortality in VHL patients. Early diagnosis and prompt treatment can mitigate tumor-related complications, improving survival and prognosis.

Regarding the molecular genetics of VHL disease, extensive literature reviews have identified the VHL gene as a tumor suppressor located on chromosome 3p25-p26. Mutations, deletions, translocations, or hypermethylation of this gene result in tumor-like changes in various organs, including the brain, pancreas, adrenal glands, kidneys, and reproductive system. The VHL gene encodes a protein that forms a complex with Elongin C, Elongin B, and Cullin 2 (CUL2), known as the VCB-CUL2 complex or E3 ubiquitin ligase. This complex activates the ubiquitin-proteasome system, facilitating the degradation of HIF subunits HIF1α and HIF2α. When VHL is mutated, HIFα subunits are stabilized, leading to the upregulation of HIF target genes involved in angiogenesis, such as vascular endothelial growth factor (VEGF), platelet-derived growth factor, transforming growth factor, glucose transporter 1, and phosphofructokinase-1. These factors promote abnormal cell division, tumor proliferation, and excessive blood vessel growth, driving tumor development (11, 14). Consequently, targeting drugs that block HIF1α and HIF2α may offer a promising approach to halt tumor growth at the root cause of VHL disease.

Treatment and management strategies for VHL-deficient RCC can be divided into three categories: active monitoring, surgical intervention, and systemic treatment.

In VHL patients with RCC, tumors are typically bilateral, multiple, and progress slowly. The average relative growth rate (RGR) is approximately 0.42%, with tumor size increasing at a rate of less than 0.3 cm per year. In cases where the initial lesion is unilateral, 1-2% of patients may eventually develop bilateral lesions (15, 16). When the tumor diameter is less than 3 cm, active monitoring is recommended. The monitoring schedule typically involves CT, MRI, or ultrasound examinations at 3 and 6 months, followed by biannual imaging until disease progression is observed. If progression occurs, immediate intervention should be implemented. If the disease remains stable for one year, annual monitoring is recommended thereafter. For patients who do not qualify for active monitoring or who show signs of progression (such as increased tumor volume or metastatic lesions on imaging), clinical treatment should be initiated based on the treatment plan (10).

For VHL patients with RCC, when the tumor diameter exceeds 3 cm, metastasis is highly likely, making surgical intervention necessary. Nephron-sparing surgery (NSS) is primarily used for treating small renal masses (SRMs) and is the preferred approach for patients with bilateral multifocal (BMF) renal tumors, solitary kidneys, or hereditary RCC syndromes. In the case of bilateral RCC associated with VHL disease, NSS is recommended as the preferred surgical method, with the surgical sequence starting with the simpler lesions and progressing to more challenging ones.

There is debate regarding whether the surgery should be performed simultaneously or in stages. Most researchers (17) suggest that for VHL patients with good physical fitness and a low ECOG score, staged surgery for bilateral RCC is appropriate. However, studies by researchers like Jacobs (18) have shown that simultaneous bilateral tumor resection can be both safe and feasible, provided the patient’s physical condition is adequate. This approach may shorten recovery time and reduce the risk of kidney disease.

For patients with poor physical conditions who cannot tolerate surgical treatment, or for those with small tumors (less than 3 cm) located relatively superficially, ultrasound and/or CT-guided RFA has proven to be an effective minimally invasive treatment with good anti-tumor efficacy. In Case Report 1, the patient had bilateral VHL-deficient RCC, with the left kidney tumor being larger. For the left kidney tumor, the treatment plan included first-stage RFA under ultrasound guidance, targeting a tumor measuring 5 cm × 6 cm (cT1b). This approach minimized the resection of healthy renal tissue, reduced surgical trauma, and yielded favorable results. For the second stage, laparoscopic resection of the left renal tumor was performed, followed by targeted drug therapy. The subsequent re-examination showed excellent outcomes (**Figure 2**). For VHL-associated RCC with larger tumors, this surgical approach should be carefully considered.

A literature review on similar cases indicates that 40% of RCC patients have tumors in the 4–7 cm range (T1b) (19–21). For T1b RCC, RFA is a viable option alongside nephron-sparing surgery. Several studies have suggested that for clinical stage T1b (cT1b) RCC, thermal ablation may provide oncological outcomes similar to nephrectomy while better preserving renal function (22–26). Jose Pablo Pedraza-Sanchez and colleagues (27) found no significant differences in efficacy, safety, or oncological outcomes between percutaneous RFA (PRFA) and PN for treating SRMs. However, the limited sample sizes and single-center nature of these studies make the clinical efficacy of thermal ablation for cT1b RCC uncertain. Additionally, while both radiofrequency and cryoablation are available, it remains unclear which method is superior for SRMs and cT1b RCC.

Active systemic therapy is critical for improving the prognosis and survival of patients with VHL combined with bilateral RCC. With the advent of combined targeted and immunotherapy, significant progress has been made in the treatment of VHL-related bilateral RCC, supported by multi-center studies and extensive clinical experience. Although reports are limited, the role of the VHL gene as a tumor suppressor is well-established. Mutation of the VHL gene disrupts its binding to HIF1α and HIF2α, preventing activation of the ubiquitin-proteasome system. This leads to the stabilization of HIFs, which in turn upregulate target genes that stimulate neo-vascularization (e.g., VEGF, platelet-derived growth factor, transforming growth factor, glucose transporter 1, and phosphofructokinase-1). These factors are involved in the growth, survival, proliferation, differentiation, and migration of RCC cells, making them key drivers of VHL-deficient tumors and important targets for tumor growth and metastasis. With the development of various targeted therapies, patients with VHL combined with bilateral RCC can now be treated using a stratified approach based on the International Metastatic Renal Cell Carcinoma Database Consortium (IMDC) criteria. First-line targeted drugs such as sorafenib, sunitinib, pazopanib, and cabozantinib have proven effective. In Patient Case 1, a relatively complex treatment regimen was employed, including RFA, laparoscopic PN, and targeted therapy with pazopanib. This patient has been under follow-up for 4 years. In Case 2, pazopanib was selected as the first-line treatment, and after 3 months, the therapeutic effect was found to be favorable. Adverse events (AEs) for both patients were limited to grade 1/2 symptoms, including hair changes, hand-foot syndrome, abdominal distension, and abdominal pain. These AEs were manageable and preventable, and treatment continuation was recommended. Follow-up for these patients is ongoing, with 10 months of observation for Case 2.

Belzutifan, an HIF-2α inhibitor, received U.S. Food and Drug Administration (FDA) approval on August 13, 2021, as a systemic therapy for VHL-deficient tumors, offering a new treatment option for patients with this rare disease. Several studies (28–32) have shown that Belzutifan has a favorable risk-benefit profile in treating RCC, central nervous system hemangioblastomas, and pancreatic neuroendocrine tumors associated with VHL disease. Belzutifan was officially launched in China on May 22, 2025. However, there is limited data on its use and follow-up in China. To fully understand its role in the treatment of VHL-associated RCC, long-term follow-up and additional clinical trials are needed.

The mechanism of action of immune checkpoint inhibitors (ICIs) primarily aims to normalize the immune system. This is achieved through two main processes (1): downregulating PD-1 expression on T cells, thereby alleviating the inhibitory effect of PD-1 on T cell activity, and (2) blocking the PD-1/PD-L1 interaction, disrupting the PD-1 signaling pathway. Immune checkpoints are critical components of the immune system, playing a critical role in preventing autoimmune diseases. However, in the context of immune evasion by tumors, the efficacy of ICIs may vary depending on the expression levels of PD-L1. Nonetheless, in principle, ICIs are effective for the treatment of tumors.

**Figure 4:**
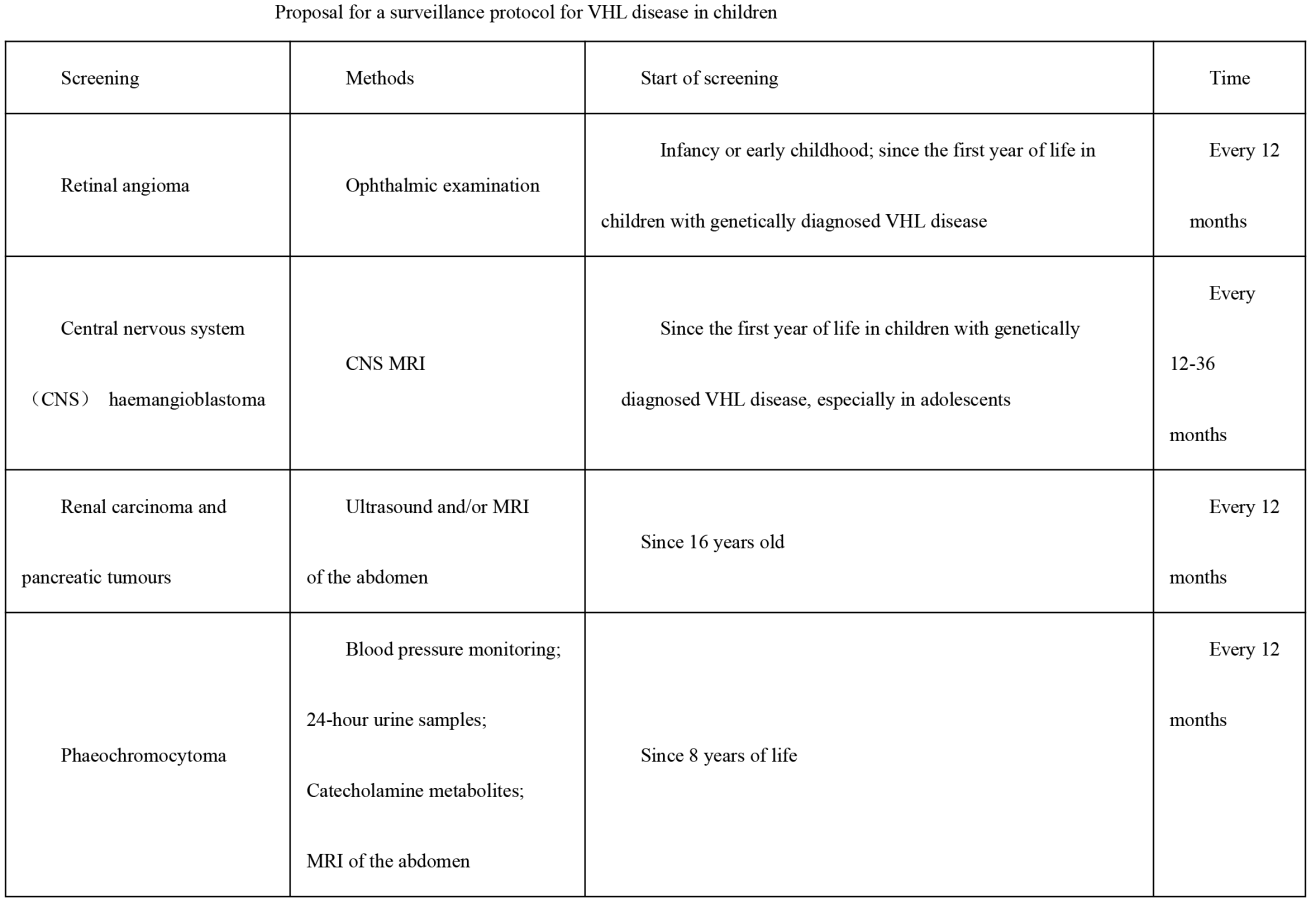
Proposal for a surveillance protocol for VHL disease in children.

As VHL disease involves multiple organs and systems, the onset of tumors in different systems occurs in varying sequences, making comprehensive tumor treatment challenging. Therefore, managing these patients requires an MDT to assess, consult, and evaluate therapeutic efficacy. The consultation of an MDT, encompassing specialists from neurology, urology, pathology, ophthalmology, otolaryngology, endocrinology, and other fields, is essential for diagnosing and treating this disease. Furthermore, early comprehensive screening and follow-up of both patients and their close family members are essential. Early detection and diagnosis contribute to improved prognosis. Recent literature suggests monitoring strategies for children with VHL disease and individuals with positive VHL mutations but no clinical manifestations (8, 33–35), as summarized in **Figure 4**.

## 5 Conclusion

VHL disease is a rare familial autosomal dominant genetic disorder. Due to the familial clustering of VHL-related lesions across multiple systems and organs, a multidisciplinary, comprehensive treatment approach is vital for managing patients. This approach better facilitates monitoring the progression of both benign and malignant lesions associated with the disease. The rapid advancements in imaging and genetic diagnostic techniques have significantly improved the early detection rates of VHL disease. Clinicians, in addition to guiding treatment, must also focus on educating patients and their families, providing screening and monitoring services, and raising awareness of the disease. Genetic screening and early imaging diagnosis are instrumental for family planning. Long-term follow-up is essential to monitor the risk of tumor development. While treatment options for VHL-deficient RCC are increasingly diverse, the pathogenesis remains an area of ongoing research, with new tumorigenic pathways continuously emerging. In the future, this will lead to more therapeutic targets and treatment plans, enhancing the personalized management of VHL-RCC patients and advancing the understanding, diagnosis, and treatment of this disease.

## Data Availability

The original contributions presented in the study are included in the article/supplementary material. Further inquiries can be directed to the corresponding authors.
